# Supplementary material for: Mapping Attenuation Determinants in Enterovirus-D68
Source: Viruses. 2020 Aug 8;12(8):867. doi: 10.3390/v12080867 (PMC7472100; doi:10.3390/v12080867)
Supplement: Supplementary file 1 [file viruses-12-00867-s001.zip › Supplementary/Data S2 polypeptide alignment.pdf.pdf]

```
                                        20          40          60          80
49129-polypeptide MGAQVTRQQT GTHENANIAT NGSHITYNQI NFYKDSYAAS ASKQDFSQDP SKFTEPVVEG LKAGAPVLKS PSAEACGYSD 80
49131-polypeptide .......... .......... .......... .......... .......... .......... ....V..... .......... 80
 4231-polypeptide .......... .......... .......... .......... .......... .......... ....V..... .......... 80
49130-polypeptide .......... .......... .......... .......... .......... .......... .......... .......... 80
                                       100         120         140         160
49129-polypeptide RVLQLKLGNS AIVTQEAANY CCAYGEWPNY LPDHEAVAID KPTQPETATD RFYTLKSVKW ETGSTGWWWK LPDALNNIGM 160
49131-polypeptide .......... .......... .......... .......... .......... .....R.... .A........ .......... 160
 4231-polypeptide .......... .......... .......... .......... .......... .....R.... .A........ .......... 160
49130-polypeptide .......... .......... .......... .......... .......... .......... .......... .......... 160
                                       180         200         220         240
49129-polypeptide FGQNVQHHYL YRSGFLIHVQ CNATKFHQGA LLVVAIPEHQ RGAHNTNTSP GFDDIMKGEE GGTFNHPYVL DDGTSLACAT 240
49131-polypeptide .......... .......... .......... .......... .......... .......... .......... .......... 240
 4231-polypeptide .......... .......... .......... .......... .......... .......... .......... .......... 240
49130-polypeptide .......... .......... .......... .......... .......... .......... .......... .......... 240
                                       260         280         300         320
49129-polypeptide IFPHQWINLR TNNSATIVLP WMNAAPMDFP LRHNQWTLAI IPVVPLGTRT TSSMVPITVS IAPMCCEFNG LRHAITQGVP 320
49131-polypeptide .......... .......... .......... .......... .......... M......... .......... .......... 320
 4231-polypeptide .......... .......... .......... .......... .......... M......... .......... .......... 320
49130-polypeptide .......... .......... .......... .......... .......... .......... .......... .......... 320
                                       340         360         380         400
49129-polypeptide TYLLPGSGQF LTTDDHSSAP ALPCFNPTPE MHIPGQVRNM LEVVQVESMM EINNTESAVG MERLKVDISA LTDVDQLLFN 400
49131-polypeptide .......... .......... V......... .......... .......... .......... .......... .......... 400
 4231-polypeptide .......... .......... V......... .......H.. .......... .......... .......... .......... 400
49130-polypeptide .......... .......... .......... .......... .......... .......... .......... .......... 400
                                       420         440         460         480
49129-polypeptide IPLDIQLDGP LRNTLVGNIS RYYTHWSGSL EMTFMFCGSF MATGKLILCY TPPGGSCPTT RETAMLGTHI VWDFGLQSSV 480
49131-polypeptide .......... .......... .......... .......... .......... .......... .........V .......... 480
 4231-polypeptide .......... .......... .......... .......... .......... .......... .......... .......... 480
49130-polypeptide ....V..... .......... .......... .......... .......... .......... .......... .......... 480
                                       500         520         540         560
49129-polypeptide TLIIPWISGS HYRMFNNDAK STNANVGYVT CFMQTNLIVP SESSDTCSLI GFIAAKNDFS LRLMRDSPDI GQLDHLHAAE 560
49131-polypeptide .......... .......... .......... .......... .......... ......D... .......... ...N...... 560
 4231-polypeptide .......... .......... .......... .......... .......... ......D... .......... ..I....... 560
49130-polypeptide .......... .......... .......... .......... .......... ......D... .......... ..P....... 560
                                       580         600         620         640
49129-polypeptide AAYQIESIIK TATDTVKSEI NAELGVVPSL NAVETGVTSN TEPEEAIQTR TVINQHGVSE TLVENFLSRA ALVSKRSFEY 640
49131-polypeptide .......... .......... .......... ......A... .......... .......... .......... .......... 640
 4231-polypeptide .......... .......... .......... ......A... .......... .......... .......... .......... 640
49130-polypeptide .......... .......... .......... ......A... .......... .......... .......... .......... 640
                                       660         680         700         720
49129-polypeptide KDHTSSTARA DKNFFKWTIN TRSFVQLRRK LELFTYLRFD AEITILTTVA VNGSGNNTYV GLPDLTLQAM FVPTGALTPE 720
49131-polypeptide ......A.QT .......... .......... .......... .......... ....S..... .......... .......... 720
 4231-polypeptide ......A.Q. .......... .......... .......... .......... ....S..... .......... .......... 720
49130-polypeptide ......A.Q. .......... .......... .......... .......... ....S....A .......... .......... 720
                                       740         760         780         800
49129-polypeptide KQDSFHWQSG SNASVFFKIS DPPARITIPF MCINSAYSVF YDGFAGFEKN GLYGINPADT IGNLCVRIVN EHQPVGFTVT 800
49131-polypeptide .......... .......... .....M.... .......... .........S .......... .......... .......... 800
 4231-polypeptide .......... .......... .....M.... .......... .........S .......... .......... .......... 800
49130-polypeptide .......... .......... .......... .......... .......... .......... .......... .......... 800
                                       820         840         860         880
49129-polypeptide VRVYMKPKHI KAWAPRPPRT LPYMSIANAN YKGKQRAPNA LSAIIGNRDS VKTMPHNIVN TGPGFGGVFV GSFKIINYHL 880
49131-polypeptide .......... .......... .......... ....G..... .N........ .........T .......... .......... 880
 4231-polypeptide .......... .......... .......... ....K..... .N........ .........T .......... .......... 880
49130-polypeptide .......... .......... .......... ...RE..... .......... .......... .......... .......... 880
                                       900         920         940         960
49129-polypeptide ATTEERQSAI YVDWQSDVLV TPIAAHGRHQ IARCKCNTGV YYCRHKNRSY PICFEGPGIQ WIEQNEYYPA RYQTNVLLAV 960
49131-polypeptide .....K.... .......I.. .......... .......... ......D... .......... ....S..... .......... 960
 4231-polypeptide .......... .......I.. .......... .......... ......D... .......... ....S..... .......... 960
49130-polypeptide ..A....... .......... .......... .......... .......... .......... .......... .......... 960
                                       980       1,000       1,020       1,040
49129-polypeptide GPAEAGDCGG LLVCPHGVIG LLTAGGGGIV AFTDIRNLLW LDTDAMEQGI TDYIQNLGNA FGAGFTETIS NKAKEVQDML 1040
49131-polypeptide .......... .......... .......... .......... ....V..... .......... .......... .......... 1040
 4231-polypeptide .......... .......... .......... .......... ....V..... .......... .......... .......N.. 1040
49130-polypeptide .......... .......... .......... .......... .......... .......... .......... .......... 1040
                                     1,060       1,080       1,100       1,120
49129-polypeptide IGESSLLEKL LKALIKIISA LVIVIRNSED LVTVTATLAL LGCHDSPWSY LKQKVCSYLG IPYVPRQGES WLKKFTEACN 1120
49131-polypeptide .......... .......... .......... .......... .......... .......... .......S.. .......... 1120
 4231-polypeptide .......... .......... .......... .......... .......... .......... .......S.. .......... 1120
49130-polypeptide .......... .......... .......... .......... .......... .......... .......... .......... 1120
                                     1,140       1,160       1,180       1,200
49129-polypeptide ALRGLDWLSQ KIDKFINWLK TKILPEAREK YEFVQRLKQL PVIENQVSTI EHSCPTTEQQ QALFNNVQYY SHYCRKYAPL 1200
49131-polypeptide .......... .......... N......... .......... .......... .......... .......... .......... 1200
 4231-polypeptide .......... .......... N......... .......... .......... .......... .......... .......... 1200
49130-polypeptide .......... .......... .......... .......... .......... .......... .......... .......... 1200
```

```
                                        1,220                 1,240                 1,260                 1,280
                                            |                     |                     |                     |
49129-polypeptide  YAVEAKRVVA LEKKINNYIQ FKSKSRIEPV CLIIHGSPGT GKSVASNLIA RAITEKLGGD IYSLPPDPKY FDGYKQQTVV 1280
49131-polypeptide  ........A. .......... .......... .......... .......... .......... .......... .......... 1280
 4231-polypeptide  ........A. .......... .......... .......... .......... .......... .......... .......... 1280
49130-polypeptide  .......... .......... .......... .......... .......... .......... .......... .......... 1280

                                        1,300                 1,320                 1,340                 1,360
                                            |                     |                     |                     |
49129-polypeptide  LMDDLMQNPD GNDISMFCQM VSTVDFIPPM ASLEEKGTLY TSPFLIATTN AGSIHAPTVS DSKALSRRFK FDVDIEVTDS 1360
49131-polypeptide  .......... .......... .......... .......... .......... .......... .......... .......... 1360
 4231-polypeptide  .......... .......... .......... .......... .......... .......... .......... .......... 1360
49130-polypeptide  .......... .......... .......... .......... .......... .......... .......... .......... 1360

                                        1,380                 1,400                 1,420                 1,440
                                            |                     |                     |                     |
49129-polypeptide  YKDSNKLDMS RAVEMCKPDG CAPTNYKRCC PLICGKAIQF RDRRTNARST IDMLVTDIIK EYRTRNSTQD KLEALFQGPP 1440
49131-polypeptide  .......... .........D .......... .......... .......... .......... .......... .......... 1440
 4231-polypeptide  .......... .........D .......... .......... .......... .......... .......... .......... 1440
49130-polypeptide  .......... .......... .......... .......... .......... .......... .......... .......... 1440

                                        1,460                 1,480                 1,500                 1,520
                                            |                     |                     |                     |
49129-polypeptide  QFKEIKISVT PDTPAPDAIN DLLRSVDSQE VRDYCQKKGW IVVHPSNELI VEKHISRAFI TLQAIATFVS IAGVVYVIYK 1520
49131-polypeptide  .......... .......... .......... .......... ..I......L .......... .......... .......... 1520
 4231-polypeptide  .......... .......... .......... .......... ..I......L .....N.... .......... .......... 1520
49130-polypeptide  .......... .......... .......... .......... ...R...... .......... .......... .......... 1520

                                        1,540                 1,560                 1,580                 1,600
                                            |                     |                     |                     |
49129-polypeptide  LFAGIQGPYT GIPNPKPKVP SLRTAKVQGP GFDFAQAIMK KNTVIARTEK GEFTMLGVYD RVAVIPTHAS VGETIYINDV 1600
49131-polypeptide  .......... .......... .......... .......... .......... .......... .......... .......... 1600
 4231-polypeptide  .......... .......... .......... .......... .......... .......... .......... .......... 1600
49130-polypeptide  .......... .......... .......... .......... .......... .......... .......... .......... 1600

                                        1,620                 1,640                 1,660                 1,680
                                            |                     |                     |                     |
49129-polypeptide  ETKVLDACAL RDLTDTNLEI TIVKLDRNQK FRDIRHFLPR YEDDYNDAVL SVHTSKFPNM YIPVGQVTNY GFLNLGGTPT 1680
49131-polypeptide  .......... .......... .......... .......... .......... .......... .......... .......... 1680
 4231-polypeptide  .......... .......... .......... .......... .......... .......... .......... .......... 1680
49130-polypeptide  .......... .......... .......... .......... .......... .......... .......... .......... 1680

                                        1,700                 1,720                 1,740                 1,760
                                            |                     |                     |                     |
49129-polypeptide  HRILMYNFPT RAGQCGGVVT TTGKVIGIHV GGNGAQGFAA MLLHSYFSDT QGEIVSSEKS GVCINAPAKT KLQPSVFHQV 1760
49131-polypeptide  .......... .......... .......... .......... .......T.. .......... .......... .......... 1760
 4231-polypeptide  .......... .......... .......... .......... .......T.. .......... .......... .......... 1760
49130-polypeptide  .......... .......... .......... .......... .......T.. .......... .......... .......... 1760

                                        1,780                 1,800                 1,820                 1,840
                                            |                     |                     |                     |
49129-polypeptide  FEGSKEPAVL NPKDPRLKTD FEEAIFSKYT GNKIMLMDEY MEEAVDHYVG CLEPLDISVD PIPLESAMYG MDGLEALDLT 1840
49131-polypeptide  .......... .......... .......... .......... .......... .......... .......... .......... 1840
 4231-polypeptide  .......... .......... .......... .......... .......... .......... .......... .......... 1840
49130-polypeptide  .......... .......... .......... .......... .......... .......... .......... .......... 1840

                                        1,860                 1,880                 1,900                 1,920
                                            |                     |                     |                     |
49129-polypeptide  TSAGFPYLLQ GKKKRDIFNR HTRDTSEMTK MLEKYGVDLP FVTFVKDELR SREKVEKGKS RLIEASSLND SVAMRVAFGN 1920
49131-polypeptide  .......... .......... .....T.... .......... .......... .......... .......... .......... 1920
 4231-polypeptide  .......... .......... ..K..T.... .......... .......... .......... .......... .......... 1920
49130-polypeptide  .......... .......... .......... .......... .......... .......... .......... .......... 1920

                                        1,940                 1,960                 1,980                 2,000
                                            |                     |                     |                     |
49129-polypeptide  LYATFHNNPG TATGSAVGCD PDIFWSKIPI LLDGEIFAFD YTGYDASLSP VWFACLKKVL IKLGYTHQTS FIDYLCHSVH 2000
49131-polypeptide  ......S... .......... .......... .......... .......... .......... .......... .......... 2000
 4231-polypeptide  ......S... .......... .......... .......... .......... .......... .......... .......... 2000
49130-polypeptide  .......... .......... .......... .......... .......... .......... .......... .......... 2000

                                        2,020                 2,040                 2,060                 2,080
                                            |                     |                     |                     |
49129-polypeptide  LYKDKKYIVN GGMPSGSSGT SIFNTMINNI IIRTLLIRVY KGIDLDQFKM IAYGDDVIAS YPHKIDPGLL AEAGKQYGLV 2080
49131-polypeptide  ....R..... .......... .......... .......... .......... .......... .......... .....H.... 2080
 4231-polypeptide  ....R..... .......... .......... .......... .......... .......... .......... .....H.... 2080
49130-polypeptide  .......... .......... .......... .......... .......... .......... .......... .......... 2080

                                        2,100                 2,120                 2,140                 2,160
                                            |                     |                     |                     |
49129-polypeptide  MTPADKGTSF IDTNWENVTF LKRYFRADDQ YPFLIHPVMP MKEIHESIRW TKDPRNTQDH VRSLCYLAWH NGEEAYNEFC 2160
49131-polypeptide  .......... V......... .......... .......... .......... .......... .......... ......D... 2160
 4231-polypeptide  .......... V......... .......... ....L..... .......... .......... .......... .......... 2160
49130-polypeptide  .......... .......... .......... .......... .......... .......... .......... .......... 2160

                                        2,180
                                            |
49129-polypeptide  RKIRSVPVGR ALTLPAYSSL RRKWLDSF* 2189
49131-polypeptide  .......... .......... ......... 2189
 4231-polypeptide  .......... .......... ......... 2189
49130-polypeptide  .......... .......... ......... 2189
```
