# Supplementary material for: Mapping Attenuation Determinants in Enterovirus-D68
Source: Viruses. 2020 Aug 8;12(8):867. doi: 10.3390/v12080867 (PMC7472100; doi:10.3390/v12080867)
Supplement: Supplementary file 1 [file viruses-12-00867-s001.zip › Supplementary/Table S1.docx]

**Table S1.** Primer sequence for the construction of infectious cDNA clones

| **Primer** | **Sequence (5’-3’)** |
| --- | --- |
| **Full-length 49129** | |
| 1FEcoRI | GCCAGTGAATTC**TAATACGACTCACTATAGGG**TTAAAACAGCCTTGGGGTTGTTCCCACTCC |
| RT30SalI | GGGCATCGGTCGACTTTTTTTTTTTTTTTTTTTTTTTTTTTTTT |
| **Full-length 49130 and 49131** | |
| 1FInFus | CGGTACCCGGGGATC**TAATACGACTCACTATAGGG**TTAAAACAGCCTTGGGGTTGTTCCCACTCCAA |
| R3390 | AATCCACATAGATAGCTGACTGTCTCTC |
| 3376F | CTATCTATGTGGATTGGCAATCAGACGT |
| RT30InFus-SbfI | CGACTCTAGAGGATCGGATCCTGCAGGTTTTTTTTTTTTTTTTTTTTTTTTTTTTTT |
| 3795F | GCCAAGGAAGTGCAAGATATGCTAATTG |
| R3808 | TTGCACTTCCTTGGCTTTATTAGAAATTG |
| **5’-UTR swap** | |
| 5UTR-Fw | CGACTCACTATAGGGTTAAAACAGCCTTGGGGTTGTTCCCACTC |
| 5UTR-Rv | AACCTGAGCTCCCATTGTTAAAATTTTCAAATTAAAGTTATCAAGATGCAAGTTG |
| 5UTR-VecFw | ATGGGAGCTCAGGTTACTAGACAACAAAC |
| 5UTR-VecRv | CCCTATAGTGAGTCGTATTAGATCCCCG |
| **P1 swap** | |
| P1_Fw | TTTGAAGATTTAATAATGGGAGCTCAGGTTACCAGACAAC |
| P1_Rv | TCCAAAACCTGGGCCAGTGTTCACTATATTATGAGGCATGGTTTTG |
| P1_VecFw | GGCCCAGGTTTTGGAGGAGTTTTTG |
| P1_VecRv | TATTAAATCTTCAAATTGGAGTTATCAAAATGCAAG |
| **VP1 swap** | |
| 30VP1Fw | CCTGACATTGGACAACCAGACCATTTACATGCAGCAGAGG |
| 30VP1Rv | TCCAAAACCTGGGCCAGTGTTCACTATATTATGAGGCATGGTTTTGAC |
| 31VecFw | GGCCCAGGTTTTGGAGGAGTTTTTGTAG |
| 31VecRv | TTGTCCAATGTCAGGGCTGTCTCTCAT |
| **Point mutagenesis** | |
| 49131-107F | CCCAACGTAATTTAGAAGCTCTTAAATCAAGGCTCAATAGG |
| 49131-107R | GAGCTTCTAAATTACGTTGGGAAGGGAGATAAAACAGGC |
| 49131-648F | AAGATCCCAACTCTTATTTATCAACTTGCATTTTGATAACTCCAATTTG |
| 49131-648R | TAAATAAGAGTTGGGATCTTTCGCAATTGATTTGACAAG |
| VP3-88F | CCACTGGACGTGCAGTTGGATGGGCCACTTAGAAACAC |
| VP3-88R | CATCCAACTGCACGTCCAGTGGAATGTTAAATAACAATTGATCG |
| VP1-1F | CATTGGACAACCCAACCACTTACATGCAGCAGAGGC |
| VP1-1R | GTAAGTGGTTGGGTTGTCCAATGTCAGGGCTGTCTCTC |
| VP1-148F | CAACACATACGCCGGTCTTCCTGACTTAACACTTCAAGCA |
| VP1-148R | CAGGAAGACCGGCGTATGTGTTGTTACTACTACCATTTACTGCTAC |
| VP1-282/283F | ATTATAAAGGTAGGGAAAGAGCACCAAATGCGCTTAATGC |
| VP1-282/283R | TTGGTGCTCTTTCCCTACCTTTATAATTTGCATTTGCAATGCTCAT |
| 2A-22F | CTTAGCTACTGCCGAAGAGAAACAGTCAGCTATCTATGTGG |
| 2A-22R | GTTTCTCTTCGGCAGTAGCTAAGTGATAGTTAATTATTTTGAAAGACC |
| 3A-47F | GATTGTAATAAGACCATCAAATGAACTACTTGTGGAAAAACAC |
| 3A-47R | CATTTGATGGTCTTATTACAATCCATCCTTTCTTTTGGCAGTA |

Restriction enzyme sites are underlined; T7 promoter sequence is shown in bold.
