# Supplementary material for: Mapping Attenuation Determinants in Enterovirus-D68
Source: Viruses. 2020 Aug 8;12(8):867. doi: 10.3390/v12080867 (PMC7472100; doi:10.3390/v12080867)
Supplement: Supplementary file 1 [file viruses-12-00867-s001.zip › Supplementary/Data S1 5UTR alignment.pdf]

```
                                20                   40                   60                   80
                                 |                    |                    |                    |
49129-5UTR TTAAAACAGC CTTGGGGTTG TTCCCACTCC AAGGGCCCAC GTGGCGGCTA GTACTCTGGT ACTTCGGTAC CTTTGTACGC 80
49131-5UTR .......... .......... .......... .......... .......... .......... .......... .......... 80
 4231-5UTR .......... .......... .......... .......... .......... .......... .......... .......... 80
49130-5UTR .......... .......... .......... .......... .......... .......... .......... .......... 80

                               100                  120                  140                  160
                                 |                    |                    |                    |
49129-5UTR CTGTTTTATC TCCCTTCCCA ATGTAACTTA GAAGTTCTTA AATCAATGCT CAATAGGTGG GGCGCAAACC AGCGCTCTCA 160
49131-5UTR .......... .......... .C........ ....C..... ......G... .......... ..T....... ...A....T. 160
 4231-5UTR .......... .......... .......... ....C..... ......G... .......... A......... ........T. 160
49130-5UTR .......... .......... ......T... .......... .......... .......... .......... .......... 160

                               180                  200                  220                  240
                                 |                    |                    |                    |
49129-5UTR TGAGCAAGCA CTCCTGTCTC CCCGGTGAGG TTGTATAAAC TGTTCCCACG GTTGAAAACA ACCTATCCGT TATCCGCTAT 240
49131-5UTR ........T. .......T.. .......C.. ..A....... .......... ........T. .......... .......... 240
 4231-5UTR ........T. ..T....T.. .......C.. ..A....... .......... ........T. .......... .......... 240
49130-5UTR .......... .......... .......... .......... .......... .......... .......... .......... 240

                               260                  280                  300                  320
                                 |                    |                    |                    |
49129-5UTR AGTACTTCGA GAAACCTAGT ACCACCTTTG GATTGTTGAC GCGTTGCGCT CAGCACACTA ACCCGTGTGT AGCTTGGGTC 320
49131-5UTR .......... .......... .T........ .......... .......... .......... .......... .......... 320
 4231-5UTR .......... .......... .T........ .......... .......... .......... .......... .......... 320
49130-5UTR .......... .......... .......... .......... .......... .......... .......... .......... 320

                               340                  360                  380                  400
                                 |                    |                    |                    |
49129-5UTR GATGAGTCTG GACATACCTC ACTGGCGACA GTGGTCCAGG CTGCGTTGGC GGCCTACTCA TGGTGAAAGC CATGAGACGC 400
49131-5UTR .......... ........C. .......... .......... .......... .......... ........A. .......... 400
 4231-5UTR .......... ........C. .......... .......... .......... .......... ........A. .......... 400
49130-5UTR .......... .......... .......... .......... .......... .......... .......... .......... 400

                               420                  440                  460                  480
                                 |                    |                    |                    |
49129-5UTR TAGACATGAA CAAGGTGTGA AGAGTCTATT GAGCTACTAT AGAGTCCTCC GGCCCCTGAA TGCGGCTAAT CCTAACCATG 480
49131-5UTR .......... .......... .......... .......... .......... .......... .......... ..C....... 480
 4231-5UTR .......... .......... .......... .......... .......... .......... .......... .......... 480
49130-5UTR .......... .......... .......... .......... .......... .......... .......... .......... 480

                               500                  520                  540                  560
                                 |                    |                    |                    |
49129-5UTR GAGCAAGTGC TCACAGGCCA GTGAGTTGCT TGTCGTAATG CGCAAGTCCG TGGCGGAACC GACTACTTTG GGTGTCCGTG 560
49131-5UTR .......... ......A... .......... .......... .......... .......... .......... .......... 560
 4231-5UTR .......... ......A... .......... .......... .......... .......... .......... ....A..... 560
49130-5UTR .......... .......... .......... .......... .......... .......... .......... .......... 560

                               580                  600                  620                  640
                                 |                    |                    |                    |
49129-5UTR TTTCACTTTT TACTTTTATG ACTGCTTATG GTGACAATTT GATATTGTTA CCATTTAGCT TGTCAAATCA ATTGCAAAAG 640
49131-5UTR .......... ...CC..... .......... .......... .......... .......... .......... .....G.... 640
 4231-5UTR .......... ...CC..... .......... .......... .........G .......... .......... .......... 640
49130-5UTR .......... .......... .......... .......... .......... .......... .......... .......... 640

                               660                  680
                                 |                    |
49129-5UTR ATCCTAAATC TTATTTATCA ACTTGCATCT TGATAACTTT AATTTGAAAA TTTTAACA 698
49131-5UTR ....C..G.. .......... ........T. ........CC ........G. ...-..T. 697
 4231-5UTR .......... .......... ........T. .......... ........G. ...-..T. 697
49130-5UTR .......C.. .......... .......... .......... .......... ........ 698
```
